# Genome-Wide Identification of the *GS3* Gene Family and the Influence of Natural Variations in *BnGS3-3* on Salt and Cold Stress Tolerance in *Brassica napus*

**DOI:** 10.3390/plants14071145

**Published:** 2025-04-07

**Authors:** Ting Jin, Xiaoshuai Hao, Zhen Huang, Xingguo Zhang, Shimeng Li, Ying Yang, Weihua Long

**Affiliations:** 1 College of Rural Revitalization, Jiangsu Open University, Nanjing 210036, China; jinting@jsou.edu.cn (T.J.); yangy@jsou.edu.cn (Y.Y.); 2 College of Agronomy, Nanjing Agricultural University, Nanjing 211800, China; 2018201034@njau.edu.cn; 3 College of Agronomy, Northwest A&F University, Yangling 712100, China; huang_zhen.8@163.com; 4 College of Agronomy, Henan Agricultural University, Zhengzhou 450046, China; xingguozhang@henau.edu.cn; 5 Institute of Agriculture, Tibet Academy of Agriculture and Animal Husbandry Sciences, Lhasa 850032, China; li_shimeng90@163.com

**Keywords:** *GS3*, gene family, salt stress, low temperature stress, natural variations, *Brassica napus*

## Abstract

Saline-alkali stress and cold damage significantly impact the yield of *Brassica napus*. G proteins play a crucial role in plant resistance to abiotic stresses, and research on G proteins in *Brassica napus* (rapeseed) is still in its early stages. In this study, we employed bioinformatics tools to systematically investigate the basic physicochemical properties, phylogenetic relationships, distribution, gene structure, cis-regulatory elements, and expansion patterns of the *GS3* gene family in *Brassica napus*. Additionally, reverse transcription polymerase chain reaction (RT-PCR) was used to analyze the response of the *BnGS3-3* gene to salt and low-temperature stresses. Natural variations were found in the promoter region of *BnGS3-3*. By conducting a promoter-driven luciferase (LUC) assay, the relationship between natural variations in the *BnGS3-3* promoter and salt and cold tolerance was analyzed. Furthermore, the impact of these natural variations on flowering time, root length, and yield was explored using phenotypic data from a population. Our research results aim to provide insights into the function and molecular mechanisms of *BnGS3-3* in *Brassica napus*, and to offer valuable genetic resources for molecular breeding to improve salt and low-temperature tolerance in *Brassica napus*.

## 1. Introduction

As an ancient and conserved signal transduction system, heterotrimeric G proteins and their associated proteins are present in almost all eukaryotic organisms [1,2,3]. The components of heterotrimeric G proteins (referred to as G proteins) include three subunits: Gα, Gβ, Gγ, along with RGS proteins [2,3]. During long-term evolution, the core components of G proteins have remained largely unchanged. The typical Gα subunit (G-protein subunit alpha) in plants possesses a complete and highly conserved G1-G5 box, capable of binding and hydrolyzing GTP [4,5]. There are fewer types of Gβ in plant cells, with only one type, AGB1, detected in multiple species such as *Arabidopsis* [3], maize [6], and wheat [7]. These Gβ proteins are mainly distributed on the plasma membrane and internal membranes, highly overlapping with the localization of Gα [5]. Gγ in plant cells can be classified into three types based on structural characteristics: Type I, Type II, and Type III [5,8]. Type I Gγ typically contains 100 to 120 amino acids, features a coiled-coil domain, and harbors a conserved DPLL motif essential for binding to Gβ. Its C-terminus can be isoprenylated to ensure the localization of Type I Gγ on the plasma membrane [8], such as AGG1 [9] and AGG2 [10] in *Arabidopsis*, and AGG1 in rice [11]. The primary distinction between Type II and Type I Gγ lies in the absence of an isoprenylation site at the C-terminus of Type II [8]. The N-terminus region of Type III Gγ is similar to that of Types I and II, but its C-terminus sequence is rich in cysteines. Members of Type III Gγ include AGG3 in *Arabidopsis* [8], and DEP1 [12], *GS3* [13], and GCA2 [14] in rice. Under non-stimulatory conditions, the heterotrimeric protein formed by the binding of the three subunits localizes to the plasma membrane [2]. Physical or chemical factors can act on receptors on the plasma membrane or within the cell, which then directly or indirectly act on heterotrimeric G proteins. The individual subunits of G proteins bind to target proteins in the cytoplasm or on the plasma membrane, triggering a signal cascade that leads to signal transduction [15]. G protein-mediated signal transduction pathways are widespread in both animals and plants. These pathways are primarily initiated by G protein-coupled receptors (GPCRs) receiving external signals, which then activate the G proteins, causing the α and γ dimers to separate and transmit the signal to downstream effectors. Ultimately, this triggers a series of physiological and biochemical responses in the cell [2,3]. RGS proteins serve as desensitization factors in G-protein signal transduction by inactivating the α subunit, allowing it to reassociate with the dimer and return to the cell membrane, thereby completing a full cycle of G-protein signal transduction [16].

Currently, G proteins have been discovered in over a dozen plant species, including *Arabidopsis* [17], rice [13], soybean [18], maize [19], *Brassica napus* [20], *Solanum lycopersicum* L. [21], and potato [22]. Studies in the model plants, such as *Arabidopsis* and rice [13,17], have shown that G proteins are involved in numerous processes of plant growth and development, including seed germination [23], lateral root formation [23,24], hypocotyl growth [25], and leaf expansion [26]. They play crucial regulatory roles in plant responses to both biotic [10] and abiotic stresses [27]. In *Arabidopsis*, GPA1 interacts with CLO7, a member of the caleosin gene family, to promote seed germination [28]. *GPA1* and *AGB1* in *Arabidopsis* regulate leaf development and shape, with mutant plants *gpal* and *agb1* exhibiting round leaves [29]. *AGB1* is also involved in flower and silique development. Compared to wild-type plants, the inflorescence apex flowers in *agb1* mutants show tighter clustering, with shorter pods and rounded apices [29]. The rice G-protein γ subunit qPE_9_-1 regulates root elongation by interacting with the OsGF14b protein and the plasma membrane H^+^-ATPase required for phosphorus utilization in rice [24]. Rice DDG1/DEP2 regulates plant height by interacting with RGA1 to modulate internode and panicle elongation, which further affects leaf and leaf sheath color and rice grain shape [30].

During the stress response processes, G proteins play a crucial role in regulating transcription and maintaining metabolic homeostasis, which prepares the plant to better withstand subsequent stress, thereby enhancing its stress resistance capabilities [27]. During stress response, *GPA1* increases the sensitivity of *Arabidopsis* to salt and drought stresses. The *gpa1-4* mutant exhibits a significantly lower germination rate under osmotic stress, while under low-nitrogen conditions, it shows higher fresh weight, chlorophyll content, and total root length through direct interactions with nitrate translocator (AtNRT1.4) and autophagy-related protein (AtATG8a) [26]. The Gβ subunit protein *AGB1* plays both a facilitating and a negative role in regulating the response of *Arabidopsis* to salt and drought stress, respectively. The *agb1-2* mutant exhibits rapid senescence and leaf albinism in seedlings under salt stress conditions, while under drought stress conditions, it shows a higher survival rate compared to the wild type [26].

Through genome-wide association analysis of 352 sorghum accessions, the research team successfully localized and cloned the major alkaline tolerance gene *AT1*. This gene encodes a G protein γ subunit (Gγ) that is highly homologous to the rice grain shape regulatory gene *GS3*, and its natural truncation mutation is significantly associated with enhanced sorghum alkaline tolerance [13]. Molecular mechanism studies revealed that *AT1* negatively regulates the phosphorylation of cell membrane aquaporins *SbPIP2;1*/*2;2* and *SbPIP1;3/1;4*, thereby impeding the efflux of hydrogen peroxide (H₂O₂) and leading to the accumulation of reactive oxygen species (ROS), which increases alkaline stress sensitivity. Conversely, gene editing-mediated knockout or natural mutation of *AT1* abolishes this inhibition, restoring ROS homeostasis and significantly improving salt-alkali tolerance [13,31]. Field trials were conducted in saline-alkali soil to assess the application of the *AT1/GS3* gene in crop production. The trials found that non-functional *AT1/GS3* mutants in some monocotyledonous plants, including sorghum, millet, rice, and maize, significantly improved crop field performance in terms of biomass or yield when grown in saline-alkali soil, compared to non-genetically modified controls [13,32].

*Brassica napus*, cultivated extensively across China, Europe, North America, and other regions, stands as a pivotal oilseed crop. Nonetheless, its growth process is prone to stress [33]. The vast expanse and ubiquitous presence of saline-alkali soil worldwide, exacerbated by the escalating issue of secondary salinization, pose a formidable challenge to agricultural development, with the relentless expansion of saline-alkali soil serving as a critical constraint [13]. The variability and complexity of autumn and winter climatic conditions in temperate countries have a profound impact on the overwintering quality of winter rapeseed [34,35]. Factors such as drought induced by prolonged cold spells and the formation of thick ice crusts during snowmelt significantly hinder the overwintering capability of winter rapeseed [36]. Low temperatures and cold damage can readily diminish seed viability, result in uneven emergence, and cause frost damage to seedlings, profoundly affecting rapeseed yield and quality [34,36]. Poor overwintering can lead to a dramatic 90% reduction in winter rapeseed yield or even total yield losses [35]. Consequently, bolstering the tolerance of *Brassica napus* to environmental stress, particularly its resistance to cold and salt, and cultivating crop varieties and genotypes adapted to stress conditions hold immense practical significance.

Research on G proteins in model plants, such as *Arabidopsis* and rice, has a relatively longer history, whereas the study of G proteins in *Brassica napus* is still in its nascent stages. In this study, we systematically investigated the *GS3* gene family in *Brassica napus* using bioinformatics methods. Additionally, we employed RT-PCR to evaluate the responsiveness of the *BnGS3-3* gene under salt and low-temperature stress conditions in *Brassica napus*. Furthermore, a promoter-driven luciferase (LUC) assay was conducted to identify superior haplotypes of *BnGS3-3.* The objective of this study is to gain insights into the functional and molecular mechanisms of *BnGS3-3* in *Brassica napus* and to provide beneficial genetic resources for molecular design breeding aimed at developing salt- and cold-tolerant soybeans.

## 2. Results

### 2.1. Identification and Characterization Analysis of the GS3 Gene Family in Brassica napus

**Figure 1:**
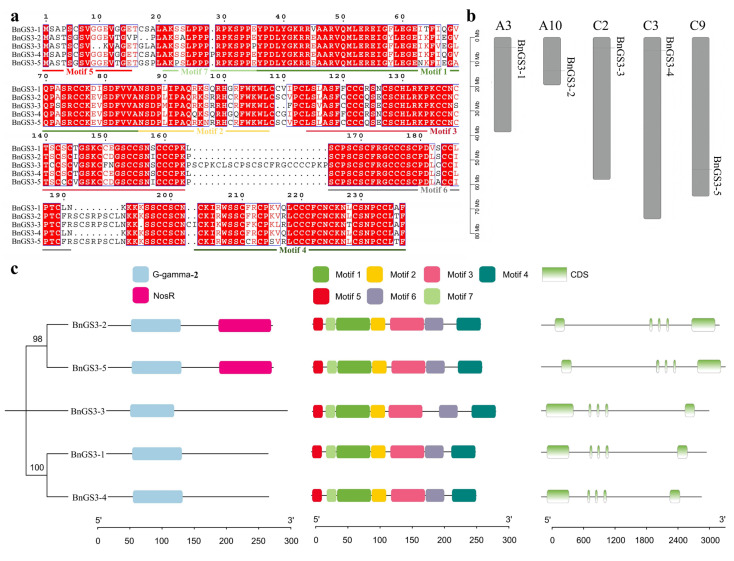
Identification of *GS3* gene family members in *Brassica napus*. (**a**) Amino acid sequences of *GS3* gene family members in *Brassica napus*, different colored lines represent the intervals of different motifs; (**b**) Chromosomal distribution of the 5 *GS3* genes in *Brassica napus*; (**c**) Analysis of the conserved domains, motifs, and gene structure of the *GS3* genes in *Brassica napus*. The unrooted tree was inferred using the MEGA X v11.0 with the Neighbor-Joining (NJ) algorithm based on BnGS3 amino acid sequences, and branch support was assessed through 1000 bootstrap replications.

In the *Brassica napus* genome, five *BnGS3* genes were discovered, sequentially named *BnGS3-1* to *BnGS3-5*. The family members consist of 237 to 265 amino acids, as shown in Figure 1a. The physicochemical properties of the BnGS3 proteins, as predicted in Table S1, reveal that their molecular weights span from 25.76 to 28.96 kDa, with isoelectric points clustered between 8 and 9. Notably, all proteins exhibit hydrophobicity indices below 0, indicating that the GS3 proteins in *Brassica napus* possess hydrophilic characteristics. In *Brassica napus*, the A and C chromosomes represent the two subgenomes resulting from the hybridization of different ancestral species. The A subgenome is derived from *Brassica rapa* and the C subgenome is derived from *Brassica oleracea*. Each subgenome carries genetic information related to the traits of specific ancestral species. The chromosomal locations of these five *BnGS3* genes are on A3, A10, C2, C3, and C9, respectively (Figure 1b), suggesting that this gene family is not organized as a gene cluster, but rather may individually perform distinct functions. Utilizing the MEME online tool to predict conserved motifs in *Brassica napus* GS3 proteins, we found that all five GS3 proteins contain the G_gamma_2 domain and seven conserved motifs, designated as Motif1 to Motif7 (Figure 1c). An analysis of the *BnGS3* gene structures revealed that all members share similar gene architectures, each comprising five exons (Figure 1c).

### 2.2. Analysis of Cis-Acting Elements in the Promoter Region of the GS3 Gene in Brassica napus

**Figure 2:**
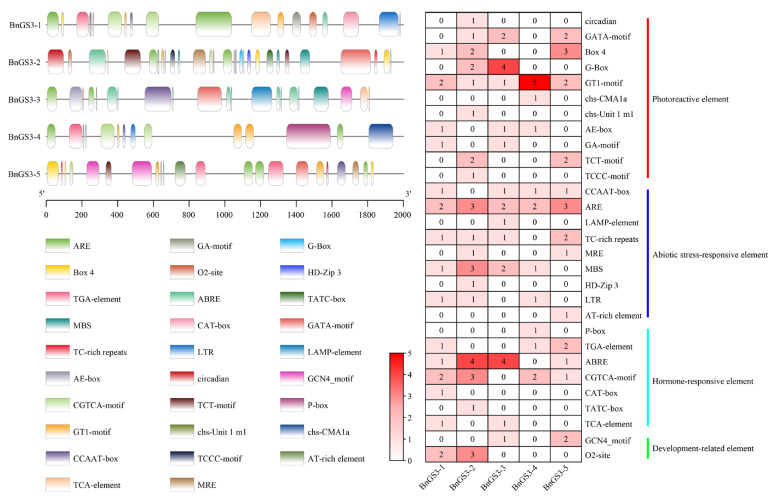
Prediction of cis-acting elements in the *GS3* gene family of *Brassica napus*. The colored boxes on the left represent different cis-acting elements, with the names of each element marked below, and the numbers on the right indicate the quantities of the corresponding cis-acting elements in the *Brassica napus GS3* genes.

Upon examining the cis-acting elements within the 2000 bp sequence upstream of the promoters of the *GS3* family genes, we identified four types of cis-acting elements present in the promoter regions of the *GS3* genes: photoreactive elements, hormone-responsive elements, abiotic stress-responsive elements, and development-related elements (Figure 2). Notably, regulatory elements associated with light response constitute the largest proportion of the promoters of *Brassica napus GS3* genes. Hormone-responsive elements primarily comprise those linked to auxin, methyl jasmonate (MeJA), abscisic acid (ABA), and salicylic acid (SA) responses. The spectrum of abiotic stress-responsive elements is diverse, encompassing hypoxia-sensitive elements, defense and stress response elements, drought resistance elements, and cold stress response elements (Table S2). These observations lead us to speculate that the *GS3* gene family in *Brassica napus* may undergo transcriptional regulation by multiple hormones and participate in a range of abiotic stress responses.

### 2.3. Expansion Pattern of the GS3 Genes in Brassica napus

**Figure 3:**
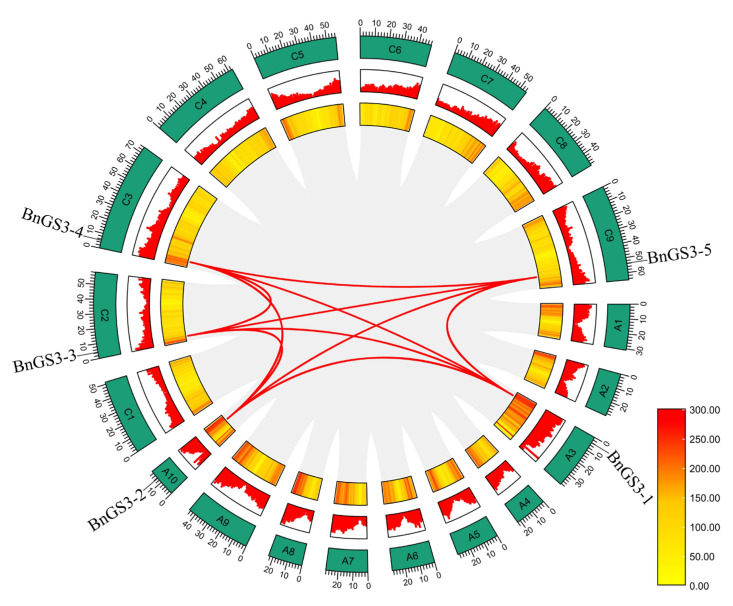
Phylogenetic analysis of the *GS3* gene family in *Brassica napus*. All syntenic blocks in the *Brassica napus* genome are depicted by gray lines, while the red lines highlight the gene pairs of the five genes in the soybean *GS3* gene family. The heatmap of genome-wide gene density, the bar chart of genome-wide gene density, and the chromosomes are demonstrated by rings from the inside to the outside, respectively. The bar in the lower right corner indicates the magnitude of gene density in the genome-wide gene density heatmap.

Collinearity analysis of the five *GS3* genes in *Brassica napus* failed to uncover any tandem duplication events among them. However, across the entire *Brassica napus* genome, ten *GS3* gene segment duplication events were identified (Figure 3). To gain insight into the differentiation mechanism of *GS3* genes during *Brassica napus* duplication events, the ratio of nonsynonymous to synonymous substitutions (Ka/Ks ratio) was calculated. The results indicated that the Ka/Ks ratios of all homologous genes between *Brassica napus* species were below 0.5 (Table S3), suggesting that these *GS3* genes have undergone intense purifying selection throughout their evolutionary history.

### 2.4. Tissue Expression Analysis of GS3 Genes in Brassica napus

**Figure 4:**
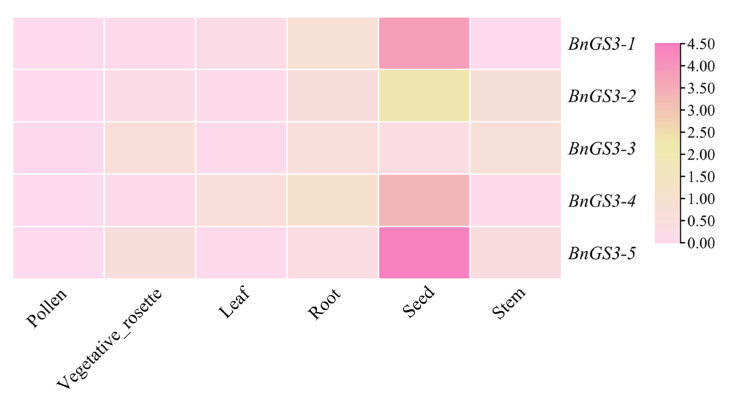
Tissue expression analysis of *GS3* genes in *Brassica napus*. The heatmap was constructed using TPM values (Transcripts Per Million) of gene expression across various tissues with TBtools v2.136. The darker the red color, the higher the expression level.

By utilizing data from the BnIR database, the expression levels of *Brassica napus GS3* gene family members were analyzed across various tissues—including roots, stems, leaves, pollen, rosettes, and seeds, as shown in Figure 4. The *GS3* genes were expressed in all tissues of *Brassica napus*, with the highest expression observed in seeds and the lowest in pollen.

### 2.5. Expression Pattern of Brassica napus GS3 Genes in Response to Low Temperature and Salt Stress Treatments

**Figure 5:**
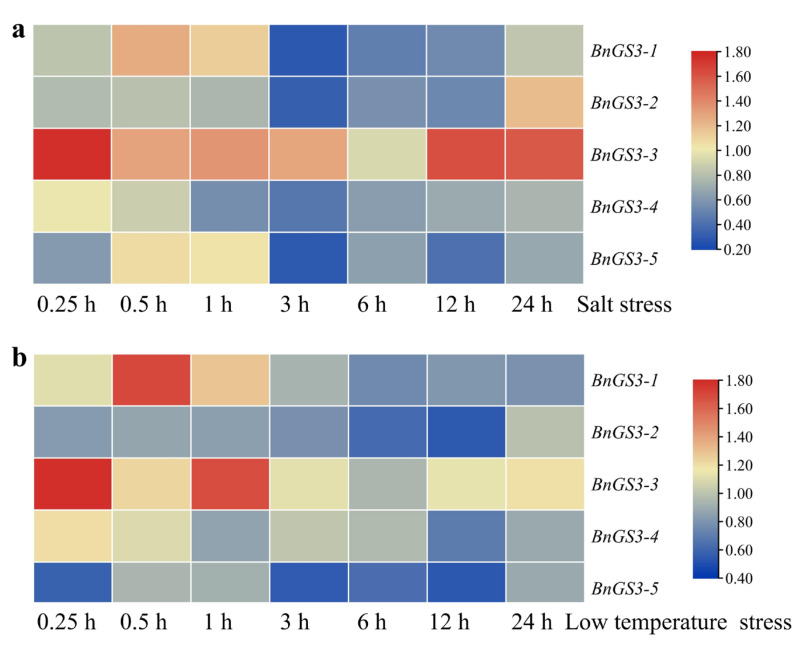
Expression heatmap of *GS3* genes in *Brassica napus* under (**a**) salt and (**b**) cold stress. The data were normalized using log_2_^(TPM-T+1/TPM-CK)^. Red: upregulated expression; Blue: downregulated expression.

To explore the role of the *Brassica napus GS3* genes in response to abiotic stress, this study employed the ZS11 online transcriptome database to analyze the expression of the *GS3* genes under conditions of salt and low-temperature stresses (Figure 5). As shown in Figure 5a, within the initial hour following salt-stress, all *GS3* genes displayed varying levels of upregulated expression. However, all *GS3* genes, except *BnGS3-3*, exhibited varying degrees of downregulation after one hour. Conversely, *BnGS3-3* showed upregulation to varying degrees within 12 h post-stress. In response to low-temperature stress (Figure 5b), *BnGS3-1*, *BnGS3-3*, and *BnGS3-4* demonstrated upregulation to differing extents within the first hour post-stress, whereas *BnGS3-2* and *BnGS3-5* consistently exhibited downregulation after low-temperature stress. Under both stress conditions, the *Brassica napus GS3* genes exhibited similar overall responses, but there were differences in the specific upregulation and downregulation patterns of individual *GS3* genes, which may be linked to their unique reaction mechanisms in response to stress. Notably, among the *Brassica napus GS3* members, only *BnGS3-3* showed upregulation to varying degrees under both salt stress and low-temperature stress.

**Figure 6:**
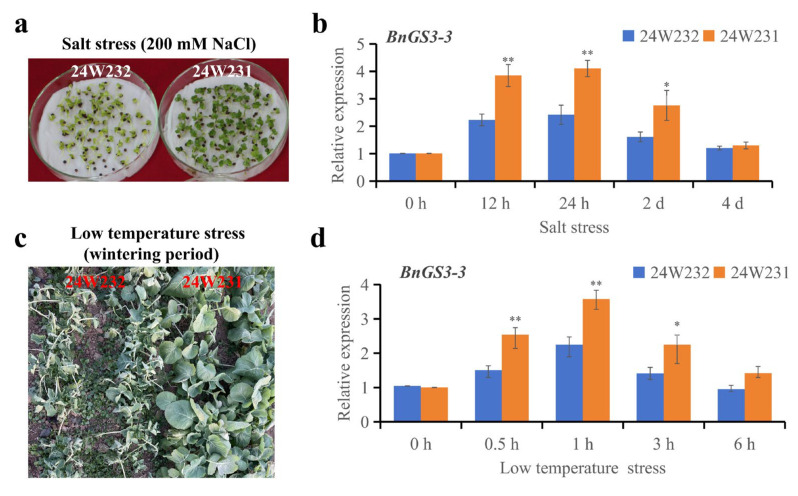
Phenotype and relative expression of *BnGS3-3* gene in two *Brassica napus* varieties under salt and cold stress. (**a**) Phenotype of two *Brassica napus* varieties after 7 days of 200 mM NaCl stress; (**b**) Relative expression of *BnGS3-3* under 200 mM NaCl stress; (**c**) Field performance of the two *Brassica napus* varieties at the 7-leaf stage during the overwintering period; (**d**) Relative expression of *BnGS3-3* under cold stress. Each treatment’s untreated group at corresponding time points was used as the control, with *BnActin2* serving as the internal reference gene. Data values represent the average of three biological replicates. Each biological replicate includes three technical replicates. Error bars indicate the standard deviation of three biological replicates. Statistical significance was analyzed using Student’s *t*-test, ** *p* < 0.01, * *p* < 0.05.

A salt-tolerant variety, 24W231, and a salt-sensitive variety, 24W232 (Figure 6a), were chosen in this study to explore the expression of the *BnGS3-3* gene in response to salt (NaCl) stress. The expression levels of the *BnGS3-3* gene in combined leaf and root samples from plants under salt stress were analyzed using quantitative reverse transcription polymerase chain reaction (qRT-PCR) (Figure 6b). The results revealed that upon exposure to salt stress (200 mmol/L NaCl), the *BnGS3-3* gene was upregulated in both varieties, peaking at the 24th hour post-stress. Notably, the relative expression levels of *BnGS3-3* in the salt-tolerant variety 24W231 were significantly higher than those in 24W232 at the 12th hour, the 24th hour, and the 2nd day post-stress. In the initial phase of this study, field evaluations were conducted to assess the cold tolerance of seven-leaf-stage *Brassica napus* seedlings during the overwintering period. Notably, it was observed that 24W231 demonstrated greater tolerance to low temperatures than 24W232 during the seedling stage in field performance (Figure 6c). When plants were subjected to low-temperature stress (4 °C) (Figure 6d), the *BnGS3-3* gene was upregulated in both varieties, reaching a peak at 1 h post-stress. Importantly, the relative expression levels of *BnGS3-3* in the low-temperature-tolerant variety 24W231 were significantly higher than those in 24W232 at 0.5 h, 1 h, and 3 days post-stress. These findings indicate that the *BnGS3-3* gene is induced in response to both salt and cold stress, potentially suggesting a role in salt and low-temperature tolerance.

### 2.6. Natural Variations in the BnGS3-3 Promoter Influence Salt and Cold Stress Tolerance in Brassica napus

**Figure 7:**
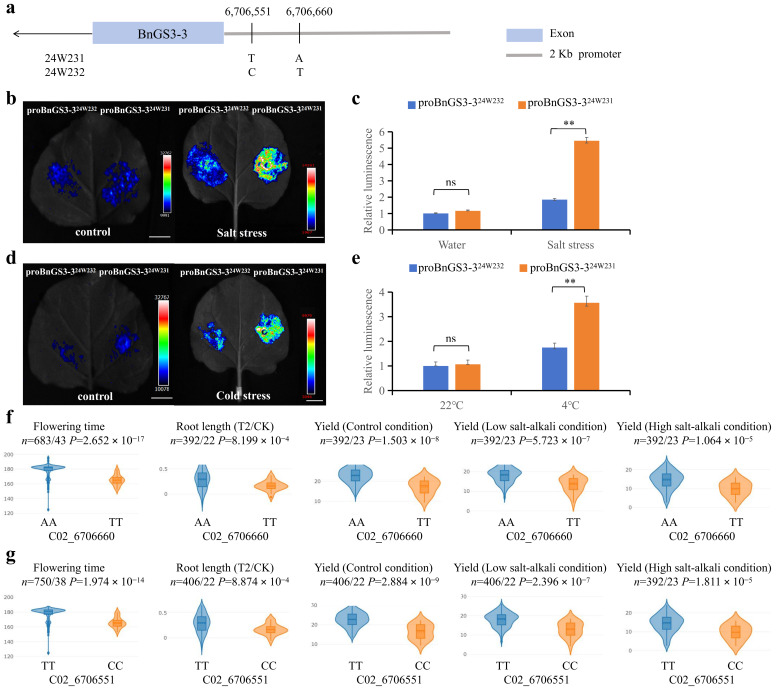
Natural variation sites in the promoter of the *BnGS3-3* gene. (**a**) Schematic diagram of the *BnGS3-3* gene promoter structure. The numbers above indicate the physical locations of the variation sites on the chromosome. (**b**,**c**) Immediate expression of activity for the two *BnGS3-3* promoter haplotypes after 16 h of 0 or 300 mM NaCl treatment. The LUC reporter gene is driven by each haplotype promoter. Photos were taken using an in vivo plant imaging system; the light intensity of b is shown in c. (**d**,**e**) Immediate expression of activity for the two *BnGS3-3* promoter haplotypes after 45 min at 22 °C or 4 °C. The LUC reporter gene is driven by each haplotype promoter. Photos were taken using an in vivo plant imaging system; the light intensity in d is shown in e. For presentation of LUC intensity values, the proBnGS3-1^24W232^ promoter haplotype under control conditions was selected as the reference control due to its minimal fluorescence intensity value. Data values represent the average of three biological replicates. Each biological replicate includes three technical replicates. Error bars indicate the standard deviation of three biological replicates. Statistical significance was analyzed using Student’s *t*-test, ** *p* < 0.01. (**f**,**g**) Boxplots of flowering time, root length, yield, yield under low-salt conditions, and yield under high-salt conditions for natural population materials carrying the natural variation sites C02_6706660 and C02_6706551. *n* refers to the number of samples carrying different haplotypes, respectively. Statistical significance was analyzed using a two-sided Wilcoxon test.

By cloning and sequencing the *BnGS3-3* promoter in 24W231 and 24W232, we identified sequence variations at 23bp and 132bp upstream of ATG, precisely located at physical positions 6706551 and 6706660 on chromosome C2 (Figure 7a). To explore the differential responses of *BnGS3-3* gene promoters from these materials to salt and low-temperature stresses, *Agrobacterium* strains harboring vectors with either pBnGS3-3^24W231^pro-GreenII-0800-LUC (proBnGS3-3^24W231^) or pBnGS3-3^24W232^pro-GreenII-0800-LUC (proBnGS3-3^24W232^) were transiently transformed into tobacco leaves (Figure 7b-e). Subsequently, the tobacco plants were exposed to salt and low-temperature treatments. Following these stresses, the area injected with the bacterial solution containing the proBnGS3-3^24W231^ vector (right side of the leaf) displayed significantly higher luciferase activity (brightness) than the area injected with the bacterial solution containing the proBnGS3-3^24W232^ vector (left side of the leaf) (Figure 7b,d). This suggests that under salt and low-temperature stresses, the *BnGS3-3* promoter in 24W231 exhibits much higher activity compared to that in 24W232.

To further quantify the differences in *BnGS3-3* promoter activity between 24W231 and 24W232 under salt and low-temperature stresses (Figure 7c,e), a luciferase activity detection kit was employed to determine the relative luciferase activity using the LUC/REN ratio. The findings revealed that, under these stress conditions, the *BnGS3-3* promoter activity in 24W231 was significantly elevated compared to that in 24W232 (*p* < 0.01).

Through an analysis of the online BnIR database, the phenotypic characteristics associated with the two natural variation sites, C02_6706551 and C02_6706660, within the population were examined (Figure 7f,g). It was observed that both variation sites correlated with flowering time, root length, and yield. Specifically, materials harboring the natural variation sites C02_6706660(A) and C02_6706551(T) (both typical of 24W231) demonstrated significantly longer flowering time, root length, and higher yield under both low and high salinity-alkalinity conditions, in contrast to materials carrying C02_6706660(T) and C02_6706551(C) (both typical of 24W232). These results imply that the *BnGS3-3* gene promoter of the 24W231 type exhibits a more pronounced response to salt and low-temperature stresses compared to the 24W232 type.

## 3. Discussion

The heterotrimeric G protein (abbreviated as G protein) complex consists of three subunits: Gα, Gβ, and Gγ, along with RGS proteins [2,3]. These components are ubiquitous in both animals and plants and have been proven to play crucial roles in plant growth and development, as well as in resistance to biotic and abiotic stresses [13,27]. Thus far, research on heterotrimeric G proteins has been conducted in crops such as *Arabidopsis*, rice, maize, and sorghum [13,17,18,19], but there have been limited reports on their presence in *Brassica napus*. In this study, we conducted a genome-wide identification and analysis of genes encoding the Gγ subunit protein GS3 [13] in *Brassica napus*, leading to the identification of five *BnGS3* genes, named *BnGS3-1* to *BnGS3-5* (Figure 1). The evolution of the *GS3* gene in *Brassica napus* is relatively conserved, with the five *GS3* members exhibiting similar conserved motifs and gene structures. All members possess the G_gamma_2 domain and seven conserved motifs (Figure 1c). A sequence alignment of the five GS3 proteins showed that *BnGS3-3* has a unique structural feature in its middle section compared to other *GS3* members, which potentially plays a pivotal role in functional differentiation (Figure 1a). In the phylogenetic tree analysis of *GS3*, *BnGS3-3* formed a separate cluster, hinting at its unique functional specificity.

During plant evolution, the processes of tandem duplication and segmental duplication frequently result in the expansion of gene families [37,38]. Ten pairs of genes demonstrate collinear relationships (Figure 3, Table S3), which may indicate their origin from chromosomal segmental duplication. These tandem and segmental duplicated genes could have contributed to the amplification of the *BnGS3* gene family in *Brassica napus*. The Ka/Ks ratio is a metric used to measure gene sequence evolution, where Ka represents the substitution rate of nonsynonymous mutations and Ks represents the substitution rate of synonymous mutations [39]. In *Brassica napus*, the Ka/Ks ratios of all homologous *GS3* genes are less than 0.5 (Table S3), indicating that these *GS3* genes have undergone strong purifying selection during evolution. Homologous genes undergoing strong purifying selection may imply that these genes play crucial roles in organisms, potentially serving as key regulatory or executive genes that are vital for growth and development, metabolism, and stress response [40,41]. The stability and functional optimization of these genes also provide organisms with better adaptability and survival advantages [39,40,41]. Therefore, the strong purifying selection experienced by these genes suggests that the *GS3* genes in *Brassica napus* play significant roles in the evolution and function of rapeseed.

Analysis of transcription data from salt and low-temperature stresses in *Brassica napus* reveals that different *BnGS3* members exhibit distinct response patterns to abiotic stresses, yet individual genes tend to respond similarly to different abiotic stresses (Figure 5). After exposure to salt and low-temperature stresses, *BnGS3-1* undergoes an initial upregulation followed by downregulation, whereas *BnGS3-3* shows upregulation to varying degrees at all post-stress time points. Conversely, *BnGS3-2*, *BnGS3-4*, and *BnGS3-5* demonstrate downregulation to varying degrees at all time points following salt and low-temperature stresses. In rice, both RGG1(I) and RGG2(I) are upregulated in response to NaCl, cold, heat, and ABA stresses, but under drought stress, only RGG1(I) is upregulated, with RGG2(I) being downregulated [42]. In *Arabidopsis*, the Gβ subunit protein AGB1 is upregulated in response to drought stress, while the Gγ subunit protein TT2 negatively regulates the response to high-temperature stress [43]. Therefore, G proteins exhibit diverse responses when confronted with abiotic stresses.

In the initial phase of this study, field evaluations were conducted to assess the cold tolerance of seven-leaf-stage *Brassica napus* seedlings during the overwintering period. This led to the identification of two distinct phenotypic materials: 24W231 (cold-resistant) and 24W232 (cold-sensitive) (Figure 6c). Notably, salt stress experiments performed during the germination stage of *Brassica napus* revealed that 24W231 exhibited a higher germination rate after salt stress compared to 24W232 (Figure 6a). Promoter cloning and sequencing of the *BnGS3-3* gene in these two materials uncovered two natural variations within their promoter sequences (Figure 7a). Analysis of the cis-acting elements within these promoters indicated that all *BnGS3* gene promoter regions contain light-responsive elements (Figure 2, Table S2), suggesting that these genes are light-inducible and may play a role in regulating the flowering time of *Brassica napus*. Early-maturing rapeseed varieties, characterized by a rapid growth cycle and early flowering, display reduced tolerance to late spring coldness. When subjected to freezing temperatures or prolonged cold and rainy conditions, rapeseed plants, particularly their delicate reproductive organs, sustain severe frost damage, impacting pollination, fertilization, and silique development. Consequently, this leads to a decline in the effective pod setting rate, seeds per pod, and seed oil content, ultimately affecting both yield and oil production [34,44,45,46,47]. By comparing the flowering times of natural population materials carrying different haplotypes of the *BnGS3-3* gene promoter (Figure 7f,g), it was found that the flowering time of materials with the 24W231 promoter haplotype (C02_6706660(A), C02_6706551(T)) was significantly longer than that of materials with the 24W232 promoter haplotype (C02_6706660(T), C02_6706551(C)). The extended flowering time conferred stronger low-temperature tolerance to materials with the 24W231 haplotype. Various hormones and stress-responsive elements exist in the *BnGS3-3* promoter region (Figure 2, Table S2). Natural variations in the promoter may affect the expression levels of *BnGS3-3* under salt and low-temperature stresses in the two materials (Figure 6). LUC activity assays of the promoter demonstrated that the 24W231 promoter of *BnGS3-3* exhibited higher activity than the 24W232 promoter under salt and low-temperature stresses (Figure 7b–e). Further validation is required to determine which specific variation(s) in the promoter region contribute to the differences in salt and cold tolerance between the two materials. Additionally, multiple cis-acting elements related to growth and development are present in the *BnGS3-3* gene promoter region (Figure 2, Table S2). G proteins play regulatory roles in processes such as seed germination [28], early seedling development [48], and root development [24]. The G-protein γ subunit qPE_9_-1 of rice interacts with OsGF14b to regulate the activity of the plasma membrane H^+^-ATPase, thereby facilitating root elongation [24]. A comparative analysis of root length among different haplotypic materials showed that the 24W231 promoter (C02_6706660(A), C02_6706551(T)) significantly enhanced root length compared to the 24W232 promoter (C02_6706660(T), C02_6706551(C)) (Figure 7f,g). It is hypothesized that *BnGS3-3* materials with the 24W231 promoter defend against abiotic stress by enhancing plant nutrition through robust root growth. Seed size is a pivotal yield trait in crops. In rice, five G-protein subunits (Gβ, Gα, DEP1, GGC2, and GS3) play a role in regulating seed size. Specifically, DEP1 and GGC2 increase grain length, whereas GS3 decreases it through competitive interaction with Gβ. By combining various G-protein variants, grain length and weight can be finely tuned, ultimately influencing rice yield [14,49,50]. A comparison of yields among natural populations carrying different *BnGS3-3* gene promoter haplotypes (Figure 7f,g) revealed that *Brassica napus* materials with the 24W231 haplotype had significantly higher yields than those with the 24W232 haplotype, and this trend remained consistent across both low and high salt environments. In summary, it is speculated that the *BnGS3-3* gene may be involved in plant flowering time, growth and development, yield, and responses to salt and low temperature. Given the environmental deterioration and increasing frequency of salt and low-temperature stresses, breeders have inadvertently selected the superior 24W231 haplotype of the *BnGS3-3* gene, which confers salt stress and low-temperature tolerance, in their efforts to breed *Brassica napus* for high yield, good quality, and stress resistance.

The functional annotation of *Brassica napus BnGS3-3* on the BnIR website specifies that it “Encodes an atypical heterotrimeric G-protein gamma-subunit involved in guard cell K^+^-channel regulation and morphological development.” G proteins can directly modulate inward potassium channels on the plasma membrane of guard cells through membrane-delimited pathways [51]. The G-protein subunits GPA1, AGB1, and AGG3 are implicated in ABA-inhibited K^+^-channel-mediated stomatal opening, with mutations disrupting this normal regulatory mechanism [52]. Potassium channels bolster plants’ resistance to abiotic stress by managing osmotic balance, maintaining ion homeostasis, influencing stomatal movement, and participating in signal transduction [53,54,55,56]. Interacting protein predictions for the *BnGS3-3*-encoded protein (Table S4) indicate that *BnGS3-3* interacts with nine proteins: AGG2, XLG2-A03, ENS-BG, XLG1, GPA1, AGG1, GB1, XLG2-C03, and GCR1. These interactions were predicted using STRING database, which has been validated and widely applied in the field to provide reliable interaction predictions [57]. Additionally, we cross-validated our predictions with experimental studies from the literature. We carefully screened and selected relevant research articles that have experimentally demonstrated interactions between similar proteins and GPCR signaling components [28,58,59,60,61]. It is hypothesized that BnGS3-3 may collaborate with these interacting proteins to contribute to stress tolerance in soybeans. GO enrichment analysis of the interacting proteins revealed that the genes encoded by these proteins are primarily involved in the G protein-coupled receptor signaling pathway, seed germination, root development, hormone response, ion transmembrane transport, etc (Table S4). The G protein-coupled receptor signaling pathway confers resistance to abiotic stresses by participating in osmotic stress response, oxidative stress response, and ion stress response [17,62]. GPA1 is involved in ABA-induced stomatal closure by regulating calcium ion concentration and reactive oxygen species (ROS) levels within guard cells, leading to stomatal closure and reduced water evaporation, thereby enhancing *Arabidopsis* tolerance to drought stress [28,58]. The guard cells lacking GPA1 exhibit defects in ABA-induced inhibition of K^+^ influx channels and pH-independent activation of anion efflux channels, disrupting the ABA signaling pathway from ABA perception to ROS production, resulting in impaired calcium channel activation [59,60]. The G protein ZmCOLD1 enhances cold tolerance in maize by regulating extracellular Ca^2+^ influx, balancing phytohormones, and interacting with other proteins [61]. Therefore, it can be inferred that BnGS3-3 may improve plant tolerance to salt and cold stress by maintaining K^+^ channel homeostasis, regulating the G protein-coupled receptor signaling pathway, and directly influencing seed germination and root growth. However, whether BnGS3 interacts with G-proteins and exerts its function through G-protein-mediated regulatory pathways remains to be experimentally verified.

## 4. Materials and Methods

### 4.1. Identification of GS3 Family Members in Brassica napus

Protein sequence files for sorghum AT1 were retrieved from the Phytozome database, while those for rice *GS3* (the ortholog of sorghum *SbAT1*) were sourced from NCBI. An analysis conducted via the Interpro online platform [63] (https://www.ebi.ac.uk/interpro/) (accessed on 2 March 2024) revealed that both transcripts of the sorghum *SbAT1* gene and the rice GS3 protein sequence possess the conserved domain G_gamma_2 (with the Interpro database ID of SM01224).

To obtain the *Brassica napus* genome data Brana_ZS11_HZAU_V1.0, it was downloaded from the NCBI database (https://www.ncbi.nlm.nih.gov/) (accessed on 10 February 2024). Subsequently, blast v2.2.9 was used to search for homologous sequences of the two transcripts of the sorghum *AT1/GS3* gene in *Brassica napus*, with a threshold evalue set at <10^−5^. The significantly aligned *Brassica napus* transcripts (evalue < 10^−5^) were further screened by querying their conserved domains one by one through the InterPro database [63]. For this purpose, resources such as InterPro [63] (https://www.ebi.ac.uk/interpro/,) (accessed on 9 March 2024), NCBI CD-Search [64] (https://www.ncbi.nlm.nih.gov/Structure/bwrpsb/bwrpsb.cgi) (accessed on 9 March 2024), and SMART [65] (https://smart.embl.de/) (accessed on 9 March 2024) were utilized to filter out sequences that contained only the G_gamma_2 conserved domain. Upon completion, duplicate transcripts belonging to the same gene were removed to finalize the *Brassica napus GS3* gene family. Finally, the genes were named based on their chromosomal location information.

The basic physicochemical properties of *Brassica napus GS3* were analyzed using TBtools v2.152 [66]. Multiple sequence alignment was performed using mega v11.0, and visualization was carried out using the ESPript 3.0 online website [67] (https://espript.ibcp.fr/ESPript/cgi-bin/ESPript.cgi) (accessed on 22 March 2024).

### 4.2. Chromosome Localization and Gene Duplication Analysis of Brassica napus GS3 Gene

To investigate the chromosomal location of *GS3* gene and its gene duplication relationships, TBtools was employed to obtain information on gene duplication. The physical chromosomal locations and gene duplication relationships of all *GS3* genes in *Brassica napus* were then visualized.

### 4.3. BnGS3 Gene Structure Analysis

The conserved motifs of the protein were analyzed using the MEME (https://meme-suite.org/meme/tools/meme) (accessed on 27 March 2024). The protein domains were analyzed online using the NCBI Web CD-Search Tool (https://www.ncbi.nlm.nih.gov/Structure/bwrpsb/bwrpsb.cgi) (accessed on 27 March 2024). The gene structure was analyzed by directly extracting the gff file. Visual analysis of the gene structure was conducted using TBtools.

### 4.4. BnGS3 Promoter Sequence Analysis

Using TBtools software, the promoter sequence located 2000 bp upstream of the transcription start site (ATG) of the *BnGS3* gene was extracted. The cis-acting elements within this sequence were predicted using PlantCARE (http://bioinformatics.psb.ugent.be/webtools/plantcare/html/) (accessed on 5 April 2024). Visual analysis of the data was conducted using TBtools.

### 4.5. Analysis of Expression Patterns of the GS3 Family Genes in Brassica napus

Expression data for *BnGS3* genes in different tissues of *Brassica napus* ZS11 and after salt and low-temperature stresses were obtained from the *Brassica napus* Integrative Resource (BnIR) online database (https://yanglab.hzau.edu.cn/BnIR) (accessed on 12 April 2024) [68,69]. The differential expression levels of *BnGS3* genes in various tissues and under salt and low-temperature stresses were analyzed and compared using log_2_(TPM-T+1/TPM-CK) [70]. Visual analysis was conducted using TBtools.

### 4.6. Salt Tolerance Test in Brassica napus Seedlings

Two *Brassica napus* germplasms with different genetic backgrounds (24W231 and 24W232) were used as materials. Uniform-sized *Brassica napus* seeds were selected, disinfected, and rinsed with distilled water before being placed in glass petri dishes (90 mm in diameter) lined with filter paper. The dishes were each added with 7.5 mL of 200 mM NaCl solution [71] and then placed in an environment with a relative humidity of 54% and a temperature of 25 °C (with a 16 h light/8 h dark cycle). Phenotypes were recorded by photography on the 7th day.

### 4.7. Material Treatment and RT-qPCR Analysis

The salt-tolerant and cold-tolerant material 24W231, along with the salt-sensitive and cold-sensitive material 24W232, were planted in pots filled with vermiculite, with each pot containing eight rape seeds. These pots were then placed in large plastic boxes and watered using 1/2 Hoagland nutrient solution. Germination took place in a growth chamber, with a controlled light/dark cycle of 16 h/8 h, temperatures maintained at 25 °C for 16 h and 20 °C for 8 h, and a light intensity set at 150 μmol m^−2^s^−1^. Once two true leaves had fully expanded, three seedlings of uniform growth were selected and retained in each pot. After three weeks of culturing, the pots were divided into three groups: a control group, a salt-stress group, and a low-temperature-stress group. The salt-stress group underwent treatment with 200 mM NaCl [71], while the low-temperature group was subjected to a temperature of 4 °C [72]. Each treatment was replicated three times to ensure accuracy. Rape seedlings were selected from the salt-stress group at various time points (12 h, 24 h, 2 d, and 4 d) and from the low-temperature-stress group at different durations (0.5 h, 1 h, 3 h, and 6 h), along with seedlings from the control group. These seedlings were ground using liquid nitrogen, and the total RNA was extracted from the rape tissue using a Plant RNA Extraction Kit (DP432, Tiangen, Beijing, China). Reverse transcription was then performed using the PrimeScript RT reagent Kit (Takara, Osaka, Japan) to synthesize cDNA. Primers were designed using NCBI-blast (Table S5), with *BnActin2* serving as the reference gene [71]. Real-time fluorescent quantitative PCR was conducted using the SYBR Premix Ex TaqTM II kit (Takara, Japan). Relative expression levels were analyzed and calculated using the 2^−ΔΔCt^ method [73].

### 4.8. Cloning and Sequencing of the BnGS3-3 Promoter

Two 7-day-old rape seedlings were selected and ground using liquid nitrogen. DNA was then extracted using a Plant DNA Extraction Kit (TransGen Biotech, Beijing, China). Based on the rape genome sequence, primers were designed online using NCBI’s primer-Blast (Table S5). Using the salt-tolerant and cold-tolerant material 24W231 and the salt-sensitive and cold-sensitive material 24W232 as templates, the *BnGS3-3* promoter was amplified. The PCR products were sequenced by Sangon Biotech Company (Shanghai, China).

### 4.9. Analysis of BnGS3-3 Promoter Activity

The 2kb promoters of the *BnGS3-3* gene, derived from material 24W231 and 24W232, were cloned into the pGreenII0800-LUC vector, respectively. The resulting expression vectors, named pBnGS3-3^24W231^pro-GreenⅡ-0800-LUC (proBnGS3-3^24W231^) and pBnGS3-3^24W232^pro-GreenⅡ-0800-LUC (proBnGS3-3^24W232^), were transformed into GV3101 cells (carrying pSoup and p19 plasmids) using the liquid nitrogen freeze-thaw method. The bacterial cells were resuspended in an injection buffer comprising 100 mmol·L⁻¹ MgCl₂, 100 mmol·L⁻¹ MES (pH = 5.7), and 150 μmol·L⁻¹ acetosyringone, with the OD600 adjusted to 0.8. The suspension was incubated in the dark at room temperature for 4 h prior to injection into the abaxial surface of 30-day-old tobacco leaves using a syringe. *Nicotiana benthamiana* seeds were sown in pots containing a 1:1 mixture of vermiculite-based nutrient soil and growth medium. Plants were subjected to transformation experiments upon reaching the five-leaf stage. After inoculation, the plants were transferred to a growth chamber for cultivation. For the salt-stress group, *Nicotiana benthamiana* plants were placed in containers filled with 300 mM NaCl treatment solution and subjected to salt stress for 16 h [74], while the low-temperature-stress group was subjected to 4 °C for 45 min [72]. LUC activity was visualized under an in vivo plant imaging system (Tanon, Shanghai, China). Additionally, luciferase activity was quantitated for each sample using a luciferase assay kit (Genscript, Promega, Nanjing, China).

### 4.10. Data Acquisition and Analysis of SNP Phenotypic Values

The yield-related phenotypic data and SNP genotypes analyzed in this study were retrieved from the BnIR database (https://yanglab.hzau.edu.cn/BnIR) (accessed on 12 April 2024). Specifically, we focused on the ZS11 cultivar, which serves as a reference genome for single-locus model analysis. The workflow is as follows: using the BnIR web interface, we applied the single-locus model to identify SNP-trait associations across candidate genes. For each SNP locus, we downloaded the corresponding phenotypic values (e.g., flowering time, yield) from the database. The violin plots (originally labeled as “box plots” in the database) and associated sample size metadata (e.g., *n* = 683/43) were directly exported from the BnIR visualization module.

## 5. Conclusions

In this study, bioinformatics methods were employed to identify five *GS3* family members in *Brassica napus*. A comprehensive analysis was then conducted, examining their basic physicochemical properties, phylogenetic relationships, distribution, gene structures, cis-acting elements, and expansion patterns. Under salt and low-temperature stresses, the five *GS3* members displayed distinct response patterns. Notably, *BnGS3-3* was up-regulated under both stress conditions, and its promoter region harbored two natural variations. When compared to *Brassica napus* germplasm materials with the 24W232 promoter, those possessing the 24W231 promoter exhibited longer flowering periods, greater main root lengths, and higher yields. Importantly, their yields remained unaffected under both low and high salt conditions, positioning *BnGs3-3* as a promising haplotype for genetic enhancement of stable yield and stress resistance in *Brassica napus*.

## Data Availability

Data are contained within the article and Supplementary Materials.

## Supplementary Materials

The following supporting information can be downloaded at: https://www.mdpi.com/article/10.3390/plants14071145/s1, Table S1: *Brassica napus GS3* genes and their encoded proteins. Table S2: Details of the cis-elements identified in this study. Table S3: Segmental duplications of *GS3* genes in *Brassica napus* and Ka/Ks ratios analysis. Table S4: The annotation of genes encoding BnGS3-3 and its predicted interacting proteins. Table S5: Primers used for qRT-PCR and vector construction.

## Abbreviations

The following abbreviations are used in this manuscript:

ABA	Abscisic acid
GPA	G-protein subunit alpha
GPCRs	G protein-coupled receptors
LUC	Luciferase
MeJA	Methyl jasmonate
ROS	Reactive oxygen species
RT-PCR	Reverse transcription polymerase chain reaction
SA	Salicylic acid
